# Dermoscopic Patterns in Juvenile Xanthogranuloma Based on the Histological Classification

**DOI:** 10.3389/fmed.2020.618946

**Published:** 2021-01-13

**Authors:** Jiaosheng Xu, Lin Ma

**Affiliations:** Department of Dermatology, National Center for Children's Health, Beijing Children's Hospital, Capital Medical University, Beijing, China

**Keywords:** juvenile xanthogranuloma, dermoscopy, clinics, histology, follow-up, diagnosis, classification

## Abstract

**Background:** Several dermoscopic features of juvenile xanthogranuloma (JXG) have been previously described in single cases or small case series and need to be further verified in a large sample.

**Objective:** We aimed to investigate the dermoscopic patterns of JXG in a large case series and the correlations of these with clinical features of different histopathological subtypes of JXG.

**Methods:** Patients who underwent dermoscopic evaluation and had a histopathological diagnosis of JXG were recruited. Histological findings, including stage and Ki67 proliferative index and the dermoscopic features of each lesion were recorded.

**Results:** Forty-one patients with JXG were included. The male to female ratio was 1.28: 1 and the median age of onset was 11 months (range: 0–95 months). Fourteen lesions were histologically categorized in the early stage, 17 in the developed stage, and 10 in the late stage. The “setting sun” pattern was observed in 35 lesions (85.4%) and “clouds” of paler yellow areas in 26 lesions (63.4%). The frequency of the “setting sun” pattern was higher in the early and developed stages (30/31) than in the late stage (5/10) (*P* = 0.002), while that of “clouds” of paler yellow areas was not significantly different between each stage. Branched linear vessels were detected in the early (11/14) and developed stage (6/17), but not in the late stage. The mean Ki67 index of the lesions with linear vessels was 11.8% (range: 2–40%), which was higher than that of lesions without linear vessels (mean index: 5%, range: 1–30%) (*P* = 0.005). The pigment network and whitish areas were only detected in 6 and 5 lesions in the late stage, respectively. The whitish areas presented either as streak or stellate shape. The pigment network exhibited either in a centric or a peripheral pattern.

**Conclusions:** The “setting sun” pattern is the characteristic dermoscopic features of JXG in the early and developed stages, while whitish areas and pigment network are the characteristic patterns in the late stage. Linear vessels present as branched patterns and mostly occur in the early stage with a high proliferative index, indicating rapid growth. The whitish areas and pigment network may present in various patterns. Dermoscopy is a useful adjunctive tool in the diagnosis and staging of JXG.

## Introduction

Juvenile xanthogranuloma (JXG) is the most common type of non-Langerhans cell histiocytosis (LCH), and is generally characterized by solitary or multiple red to yellow-brown papules or nodules on the skin (1). It is a rare histiocytic disorder mainly occurring in infancy and early childhood, but it can present in adulthood. JXG is slightly more prevalent in males than in females. The lesions are mostly confined to the skin and tend to undergo spontaneous involution within several months to years (2). Lesions are predominantly present on the head, neck, and upper trunk area. Clinically JXG presents as dome-shaped smooth papules or nodules within 2 cm in diameter. There are several clinical forms of JXG, including giant JXG (>2 cm in diameter), multiple JXG (>3 skin lesions), clustered, intraoral, intraocular, subcutaneous, and intramuscular JXG (2). Most lesions resolve without complications, while a few lead to atrophy and pigmentation at the primary location, which can cause cosmetic problems. Extracutaneous involvement is rare, but cases of systemic JXG have been reported and result in higher morbidity and mortality (3).

The histopathology of JXG shows sheets of histiocytes infiltrated with Touton giant cells and inflammation extending from the upper dermis to the subcutaneous tissues. The infiltrating histiocytes classically show the following patterns: CD68++, CD163++, Factor XIIIa+, Fascin+, S100±, CD1a–, and CD207–. Based on morphological features, JXG is classified into early JXG (irregular or folded nuclei, less vacuolated cytoplasm, and rare Touton giant cells), developed JXG (classic JXG, foamy plasma and scattered Touton giant cells), and late JXG (transitional JXG, resembling fibrous histiocytoma) in accordance with the clinical process (1). The diagnosis is usually clinical, but can be confirmed by histology in cases of diagnostic doubt. While most JXG patients do not need special treatment, systemic JXG often requires aggressive therapies similar to those used for LCH (4, 5).

Dermoscopy is a noninvasive technique used to visualize pigmented structures and vessels. Since the 1980s, dermoscopy has become widely used in the diagnosis of pigmented lesions and has been gaining popularity for its ability to differentiate between nonpigmented skin tumors and inflammatory skin diseases. It has been increasingly used in pediatric patients because it is simple, quick and noninvasive. In 2007, Antony Palmer and Jonathan Bowling first described the dermoscopic features of three patients with JXG (6). All lesions exhibited a similar “setting sun” pattern in their study. Since then, several case reports have described this classic pattern on dermoscopy and additional dermoscopic features of “clouds” of paler yellow areas, whitish areas, linear vessels, and a pigment network (7–10). Song et al. investigated 11 patients with JXG and discussed the structural correlations between dermoscopic and histopathological features of JXG (11). The “setting sun” pattern may hold diagnostic value in the early and classic stages, while the “clouds” of paler yellow areas are more common in the classic and transitional stages (12). Dermoscopy is also valuable in defining the stage of JXG patients during follow-up (13).

However, clinical reports of dermoscopic findings in a large series of patients are lacking. We investigated a series of 41 pediatric patients with JXG to explore their dermoscopic features, histopathological correlations, and to evaluate the use of dermoscopy in follow-up.

## Materials and Methods

From June 2017 to September 2019, 41 pediatric patients with histologically proven JXG at Beijing Children's Hospital, Capital Medical University, National Center for Children's Health, were included in this study with institutional review board approvals. Granulomatous inflammatory diseases, xanthoma with metabolic abnormality, sebaceous nevus, or Fordyce disease were excluded. Mastocytomas and other forms of histiocytoses, including Erdheim-Chester disease and Rosai-Dorfman disease, were also excluded by morphology and immunochemistry analysis. Written informed consent for biopsy was obtained from parents.

### Dermoscopy

Dermoscopic examination was performed using a polarized handheld dermoscope at x10 magnification (Handscopy, FotoFinder, Bad Birnbach, Germany). The dermoscopic images was captured using a high-resolution mobile camera phone (iPhone 6, Apple Inc., CA, USA) attached to the dermoscope. The following dermoscopic features were included in the study and scored as present or absent: “setting sun” sign, “clouds” of paler yellow areas, a whitish area, vascular sign, and pigment network.

### Histopathology

After dermoscopic examination, each patient underwent a punch biopsy of one well-examined skin lesion. Every lesion was examined by an experienced dermatologist. Based on the morphology of histiocytic cells, the histopathological stages were classified into an early stage (early JXG), a developed stage (classic JXG) and a late stage (transitional JXG). In the early JXG, it is characterized by the sheet-like proliferation of small monomorphous histiocytic cells. In the classic JXG, xanthomatized cells and Touton giant cells become conspicuous. In the transitional JXG, moderate fibrotic tissue reactions with increased numbers of spindle-shaped cells are prominent. All of the infiltrated histiocytes were identified by positive CD68 or CD163 immunostaining and negative CD1a, S100, and Langerin immunostaining. The proliferative index was assessed by Ki67 positive histiocytes (defined as low 1–4%; moderate 5–9%; high >10%).

### Treatment and Follow-Up

All patients were consented to treat with a watch and wait policy with regular follow-up via outpatient visit or mobile video (WeChat). The last follow-up ended in May 2020 and the mean follow-up duration was 28 months (range: 8–35 months) Clinical events were recorded.

### Statistical Analyses

Descriptive statistics for categorical variables were represented as numbers and % values. The relationship between two categorical independent variables was evaluated using the chi-square test. SPSS Windows version 24.0 package software (SPSS Inc., Chicago, IL, USA) was used for statistical analysis and *P* < 0.05 was considered statistically significant.

## Results

All patients underwent dermoscopic examination at presentation and were histologically diagnosed with JXG after a biopsy was obtained.

### Clinical and Histopathological Findings of JXG

A total of 41 patients (23 boys and 18 girls) with JXG were included. The median age at onset was 11 months (range: 0–95 months). Thirty patients had a solitary lesion (16 in the head and neck, seven in the trunk and seven in the extremities), two patients had giant JXG, and 11 patients had multiple lesions. Among patients with multiple lesions, one had both eye and liver involvement, while another had a single eye involvement. Histologically, there were 14 cases in the early stage of JXG, 17 cases in the developed stage of JXG, and 10 cases in the late stage of JXG. Low proliferative index (Ki67: 1–4%) was detected in 12 lesions (one early stage, seven developed stage, and four late stage), moderate proliferative index (Ki67: 5–9%) in nine lesions (two early stage, four developed stage, and three late stage) and high proliferative index (Ki67: >10%) in 17 lesions (11 early stage, four developed stage, and two late stage).

### Dermoscopic Features of JXG and Its Clinical and Histological Correlations

**Figure 1:**
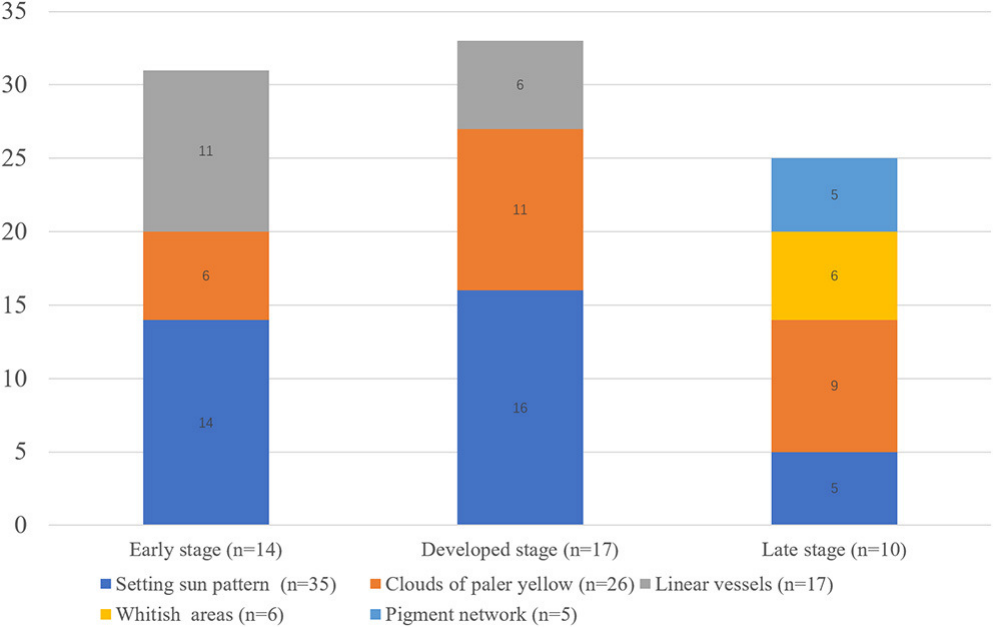
Dermoscopic feature distribution in various histological stages.

**Figure 2:**
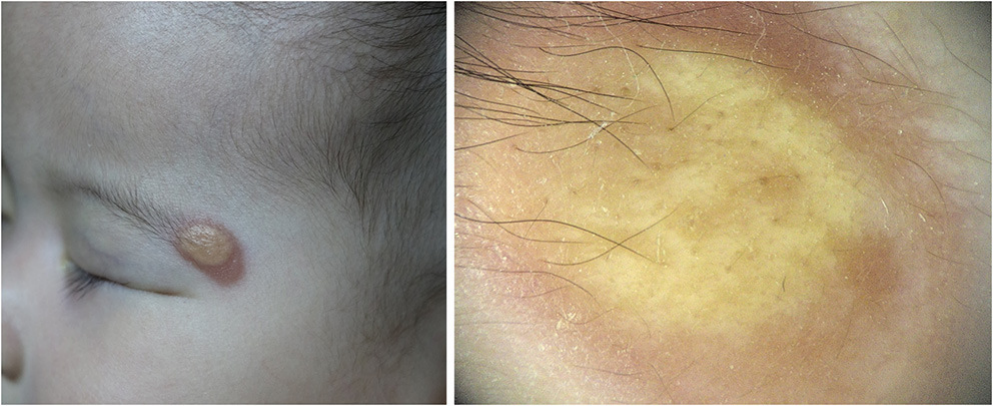
Setting sun pattern **(Right)** and its clinical appearance **(Left)**.

**Figure 3:**
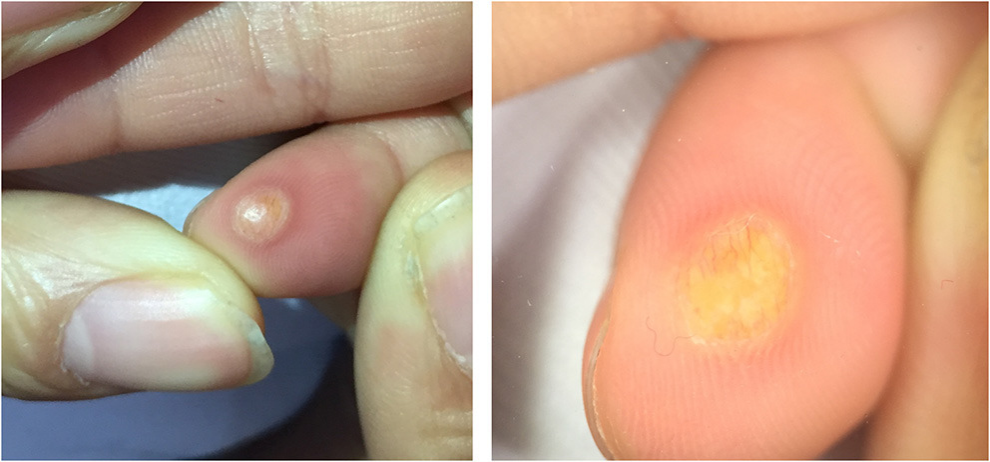
Branched linear vessels in a periphery to center pattern.

**Figure 4:**
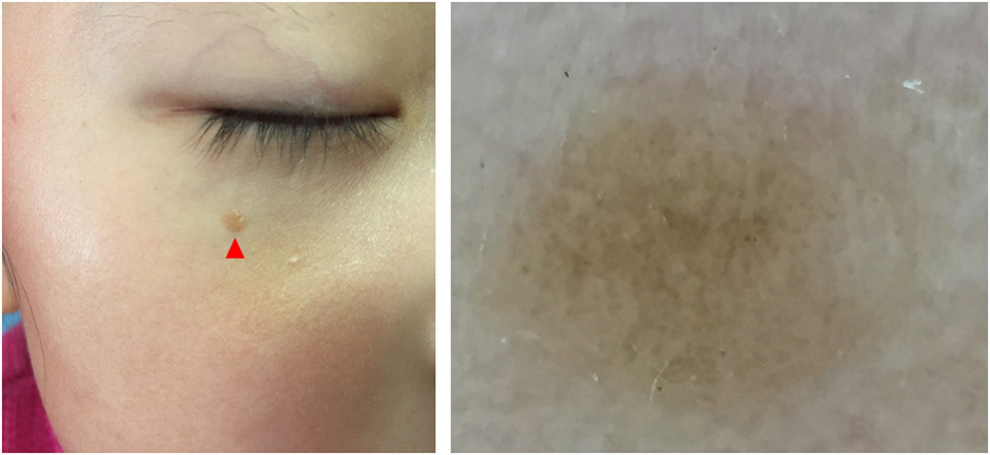
Central pigment network **(Left)** and its clinical appearance **(Right)**, arrow head.

**Figure 5:**
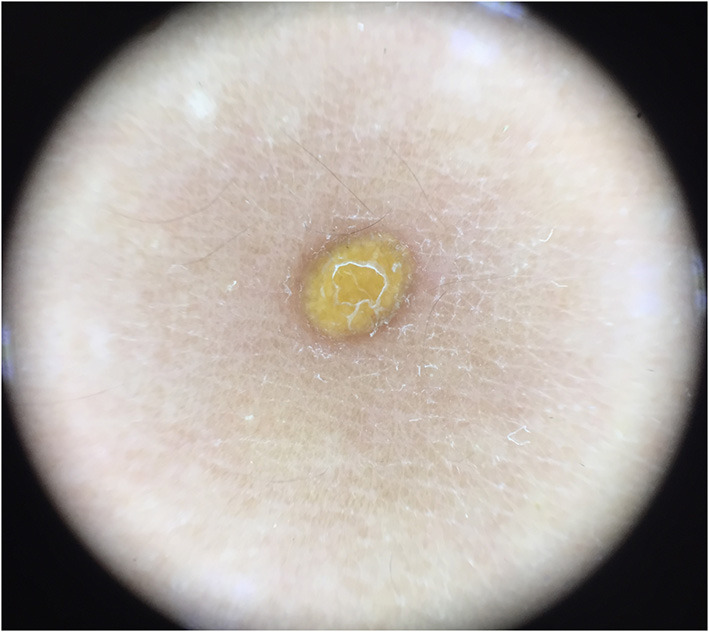
Haloed pigment around the xanthomatous lesion, showing the pigmentation in a peripheral pattern compared with Figure 4.

**Figure 6:**
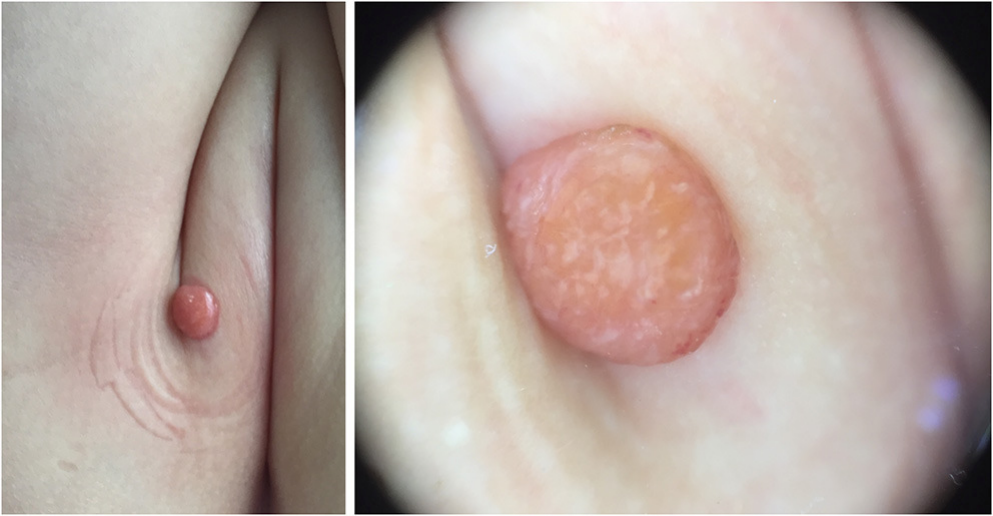
Whitish streak and coalition into small patches.

Five dermoscopic features were observed in this case series, including the “setting sun” pattern (35/41), “clouds” of paler yellow areas (26/41), linear vessels (17/41), pigment network (6/41), and whitish area (5/41) (Figure 1). The “setting sun” sign consists of a yellowish background with subtle erythema peripherally (Figure 2). The former is due to the infiltration of neoplastic histiocytes with variable lipidosis, while the latter is a vascular reaction to the parenchyma histologically. Linear vessels were present as branched from the periphery to the center (Figure 3). The pigment network exhibited either a centric or a peripheral pattern (Figures 4, 5). Whitish areas were arranged in a streak or stellate pattern and coalesced to patches in two lesions (Figures 6, **9**). Histopathological examination of JXG with whitish area revealed dermal fibrous tissue, while the pigment network represents hyperpigmentation in the basement of the epidermis.

**Figure 7:**
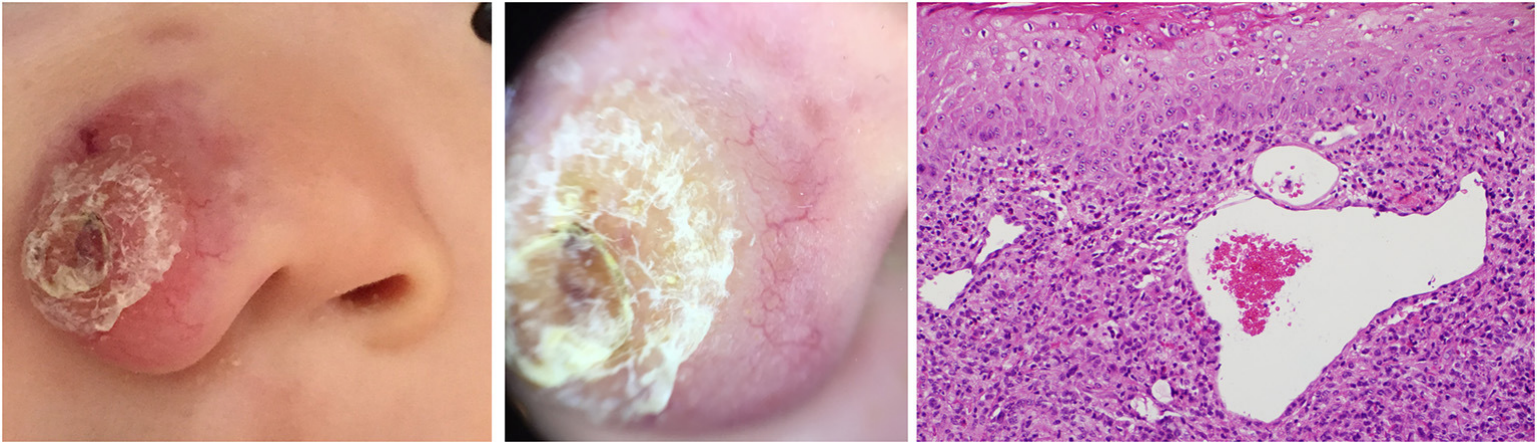
Early stage of JXG **(Right)**, H&E original magnification x20 with clinical **(Left)** and dermoscopic **(Middle)** correlations.

**Figure 8:**
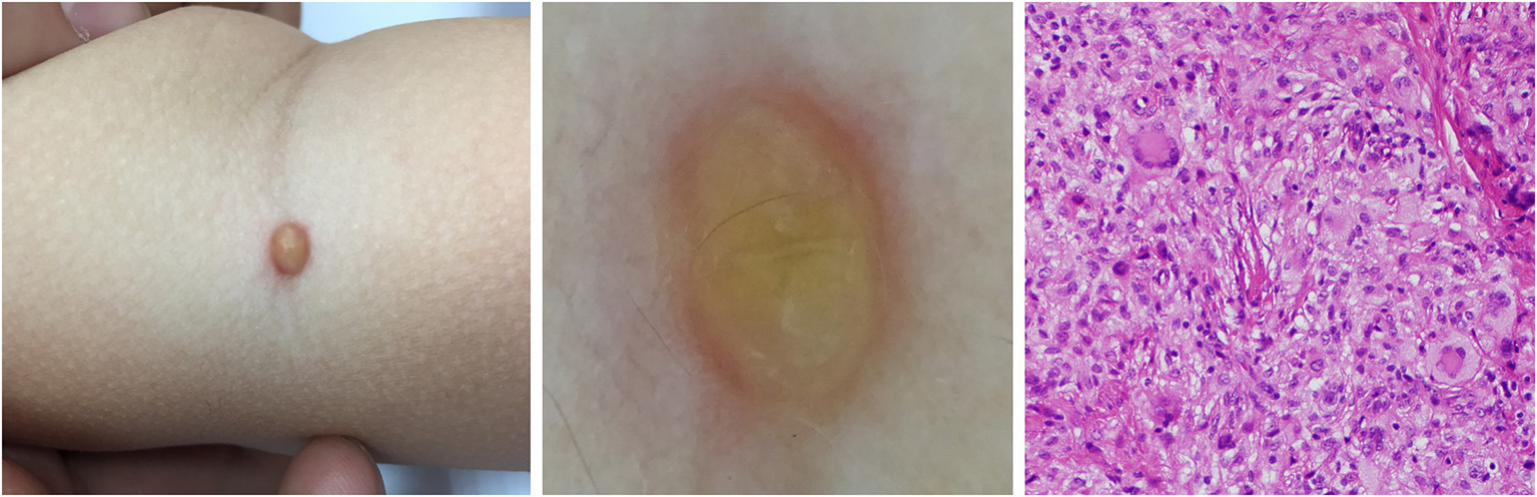
Developed stage of JXG **(Right)**, H&E original magnification x40 with clinical **(Left)** and dermoscopic **(Middle)** correlations.

**Figure 9:**
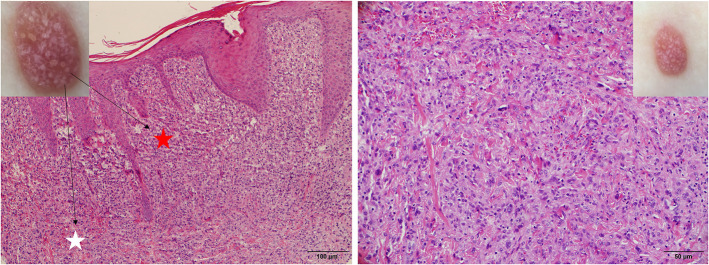
Late stage of JXG has been revealed **(Right)**, H&E original magnification x40 with clinical **(Upper Right)** correlations and stellate whitish areas and paler yellow globules **(Upper Left)** show corresponding histological manifestations (stars, H&E original magnification x20).

The “setting sun” pattern and “clouds” of paler yellow areas were found in lesions of all histological stages. The frequency of the “setting sun” pattern was higher in the early and developed stages (Figures 7, 8, 30/31) than that in the late stage (Figure 9, 5/10) (*P* = 0.002), while that of “clouds” of paler yellow areas did not differ significantly between each stage. Linear vessels were detected in the early (Figure 7) and developed stages but not in the late stage. The mean Ki67 index of the lesions with linear vessels was 11.8% (range: 2–40%), which was higher than that in lesions without linear vessels (mean index: 5%, range: 1–30%) (*P* = 0.005, Table 1). Among the 10 lesions in the late stage, the pigment network and the whitish area were detected in six and five lesions, respectively. Only one lesion in the late stage presented with both pigment network and whitish area.

**Table 1:** The proliferative index of infiltrated histiocytes in JXG with or without linear vessels.

**Linear vessels** **(*n* = 38)**	**Ki67 index**			***P*^†^**
	**Low** **(*n* = 12)**	**Moderate** **(*n* = 9)**	**High** **(*n* = 17)**	
Present (*n* = 17)	4	2	11	0.005
Absent (*n* = 21)	8	7	6	

† *Mann-Whitney U test*.

### Management and Follow-Up of JXG

The majority of patients were followed up with a wait and watch policy, while 3 patients underwent surgical resection, topical mometasone furate, and intralesional compound betamethasone, respectively, for rapid growth. At the last follow-up, the lesions showed complete resolution in 26 patients, partial resolution in 13 patients, and two patients were lost to follow-up. The mean time of complete resolution was 18 months (range: 3–34 months).

## Discussion

The dermoscopic features of JXG have been described in several single cases and small case series over the last decade, and include “setting sun” appearance, “clouds” of paler yellow areas, linear vessels, pigment network, and whitish streak. All five dermoscopic features of JXG were observed in our study. Moreover, stellate whitish areas and central pigment networks were also observed, expanding the dermoscopic patterns of JXG.

The most common dermoscopic feature of JXG observed was the “setting sun” pattern, which is in accordance with previous reports (7, 10, 14). The “setting sun” pattern is mainly present in the early and developed stages and occasionally in the late stage, and its frequency differs significantly between the late stage and the early and developed stage in this study. The “setting sun” pattern consists of a yellowish background with a peripheral capillary reaction and has also been described as a feature of reticulohistiocytoma, Erdheim-Chester disease, and xanthoma disseminatum (15). However, these disorders have their own distinctive clinical presentations that differentiate them from JXG (16). The “setting sun” pattern could aid in the diagnosis of JXG in clinical settings. Without a detailed clinical history to aid in the diagnosis of JXG, isolated dermoscopic evaluation could lead to the misdiagnosis of a lesion as an ulcerated Spitz nevus or mastocytoma with the “setting sun” pattern (17, 18).

The whitish area and the pigment network were the most frequent dermoscopic patterns in the late stage of JXG, and these patterns rarely occur in the early or developed stage. In our series, they almost exclusively presented in the late stage, and both were detected in one case. Histopathological examination of JXG with whitish area revealed dermal fibrous tissue, while the pigment network represents hyperpigmentation in the basement of the epidermis, indicating the regression of the lesions. Both peripheral and central pigment network can be observed, showing different patterns of regression of JXG. Thin whitish lines and circles in a parallel and perpendicular pattern were not observed in our study (9). Whitish areas with stellate appearance were detected in one case, which showed fibrosis in a corresponding pattern.

The pattern of “clouds” of paler yellow areas was observed in 63.4% (26/41) of cases with JXG, which was less frequent than reported by Song et al. (11). The “clouds” of paler yellow areas, which correlate with histological deposition of xanthomatized histiocytes surrounded by fibrosis, may represent the evolution of the yellow background in the “setting sun” sign.

Linear vessels were present as branched in this study. The vessels were seen on the surface of the tumors and they ran from the periphery to the center of the lesions in the majority of cases. Small comma-shaped vessels at the periphery described in adult JXG were not detected in our pediatric population of JXG (19). There was a high frequency of linear vessels in the early and developed stages, but not in the late stage. This vessel pattern suggested an episode of rapid growth as indicated by the Ki67 proliferative index. Lesions with these dermoscopic pattern are usually characterized by progressive enlargement in size, resulting in necrosis, scarring, or cosmetic complaints. Three patients with linear vessels on the face underwent rapid growth and received corresponding therapy which was successful and did not result in cosmetic side effects.

JXG is generally a self-healing disorder that regresses spontaneously during follow-up. Therefore, noninvasive clinical examination and observation is the ideal strategy in typical cases. In children, skin biopsy is avoided where possible due to the risk of pain, scarring, and psychologic trauma, especially at an early age. Based on the clinical and follow-up data, dermoscopic and histopathological findings, we propose that dermoscopy is a useful adjunctive tool in the diagnosis and staging of JXG in a clinical setting. However, cases that persist should be considered for excision biopsy. Overall, the diagnosis of JXG is based on clinical and dermoscopic findings, and biopsy may be required if there is no evidence of the late stage.

To our knowledge, this is the largest case series of JXG that investigated the dermoscopic characteristics based on histological stages. However, this study is limited by its retrospective nature and further prospective studies of larger samples should be conducted to expand upon the results described herein.

## Data Availability Statement

The original contributions presented in the study are included in the article/supplementary material, further inquiries can be directed to the corresponding author/s.

## Ethics Statement

The studies involving human participants were reviewed and approved by Institutional Review Board of Beijing Children's Hospital. Written informed consent to participate in this study was provided by the participants' legal guardian/next of kin. Written informed consent was obtained from the individual(s), and minor(s)' legal guardian/next of kin, for the publication of any potentially identifiable images or data included in this article.

## Author Contributions

JX conceptualized and designed the study, drafted the initial manuscript, reviewed and revised the manuscript, designed the data collection instruments, collected data, conducted the initial analyses. LM conceptualized and designed the study, coordinated and supervised data collection, and critically reviewed the manuscript for important intellectual content. Both authors approved the final manuscript as submitted and agree to be accountable for all aspects of the work.

## Conflict of Interest

The authors declare that the research was conducted in the absence of any commercial or financial relationships that could be construed as a potential conflict of interest.
